# National Danish surveillance of invasive clinical *Haemophilus influenzae* isolates and their resistance profile

**DOI:** 10.3389/fmicb.2023.1307261

**Published:** 2023-11-22

**Authors:** Hans-Christian Slotved, Thor Bech Johannesen, Marc Stegger, Tine Dalby, Kurt Fuursted

**Affiliations:** ^1^Department of Bacteria, Parasites and Fungi, Statens Serum Institut, Copenhagen, Denmark; ^2^Department of Infectious Disease Epidemiology & Prevention, Statens Serum Institut, Copenhagen, Denmark

**Keywords:** Denmark, epidemiology, resistance profile, *Haemophilus influenzae*, antibiotic susceptibility, serotype, COVID-19, resistance genes

## Abstract

**Introduction:**

This study aimed to investigate the epidemiology, serotype distribution, phenotypical antibiogram, and molecular resistance gene characteristics of invasive *Haemophilus influenzae* infections in Denmark from 2014 to 2022. Additionally, the potential impact of outdoor temperature and COVID-19 restrictions on the epidemiology of *H. influenzae* was assessed.

**Materials and methods:**

Invasive *H. influenzae* isolates were received from patients with positive culture results from cerebrospinal fluid, blood, or other sterile sites. Sample data were obtained from the Danish laboratory surveillance system/MiBa database, and whole-genome sequencing (WGS) was performed on the isolates. The incidence rates and distribution of *H. influenzae* cases were analyzed, and antibiotic susceptibility were assessed.

**Results:**

A total of 1,007 invasive *H. influenzae* cases were identified, with serotyping conducted for 752 (74.7%) isolates. The median incidence per year of *H. influenzae* was 2.0 cases per 100,000, with the highest incidence in 2014 and the lowest in 2020. The majority of *H. influenzae* isolates were non-typeable *H. influenzae* (NTHi), while the most prominent serotypes were serotype f followed by serotype b. Bacteremia cases accounted for the majority (88.6%) of occurrences, although meningitis cases showed an increasing trend during the time period. The age group 85+ exhibited the highest incidence. The implementation of COVID-19 preventive interventions in 2020 resulted in a significant reduction in *H. influenzae* incidence, which returned to pre-COVID levels in 2021. A negative correlation was observed between monthly *H. influenzae* cases and outdoor temperature. An overall level of genetic beta-lactamase resistance of 26.3% was observed divided into 10.6% beta-lactamase-positive ampicillin-resistant (gBLPAR), 13.6% beta-lactamase-negative ampicillin-resistant (gBLNAR) and 2.1% beta-lactamase-positive amoxicillin clavulanate-resistant (gBLPACR). Other non-beta-lactam resistance traits were detected in 7.6% of isolates (primarily aminoglycoside-modifying enzymes).

**Conclusion:**

The overall incidence of *H. influenzae* in Denmark returned to stable levels after the COVID-19 epidemic, with NTHi strains dominating. The COVID-19 preventive interventions led to a major reduction in incidence. A significant negative correlation between the incidence of *H. influenzae* and temperature was observed. The study revealed an overall genetic beta-lactam resistance rate of 26.3%, and the concordance between genotypic and phenotypic beta-lactam resistance was high (98.2%).

## 1 Introduction

*Haemophilus influenzae* is a Gram-negative bacterium that can cause a range of infections, including respiratory infections as well as invasive infections such as septicemia and meningitis in humans. *H. influenzae* is grouped into six serotypes (Hia, Hib, Hic, Hid, Hie, and Hif) according to variation of the polysaccharide capsule, whereas non-typeable *H. influenzae* strains are referred to as NTHi (Nørskov-Lauritsen, 2014; Heinz, 2018). In the pre-vaccine era, *H. influenzae* serotype b (Hib) was a frequent cause of bacterial meningitis among young children, but after the introduction of Hib vaccines in the 1990s, the numbers diminished dramatically worldwide. It has been estimated that in 2000, Hib caused more than eight million cases of pneumonia, meningitis, and other severe diseases and more than 370,000 deaths globally in children less than 5 years (Watt et al., 2009).

The Hib vaccine was highly successfully (96% 12-month vaccination coverage) introduced into the Danish immunization program in 1993 as a part of the Diphtheria-Tetanus-Pertussis-Polio combination vaccine recommended at three, five, and 12 months of age,^1^ and has been estimated to have an effectiveness of more than 97% for the prevention of Hib-related meningitis.^2^

Surveillance of Danish invasive *H. influenzae* cases and together with phenotypic susceptibility to beta-lactams has been reported in the Danish Integrated Antimicrobial Resistance Monitoring and Research Programme (DANMAP) since 2017.^3^ Resistance toward beta-lactams in *H. influenzae* in Danish invasive strains has been high and consistent with phenotypic resistance toward penicillin up to 31% (Birgitte Borck Høg, 2021). The molecular antibiotic resistance in *H. influenzae* is more diverse, especially in the beta-lactam antibiotic group where both beta-lactamase production or alterations in penicillin-binding proteins (PBPs), particularly PBP3 encoded by the *ftsI* gene, leads to resistance to beta-lactam antibiotics. Resistance toward the quinolones is still rare, although high-level ciprofloxacin resistance had been described in Denmark (Fuursted et al., 2016). Surveillance of serotype distribution and prevalence of drug-resistant strains in the general population is critical to maintain appropriate prevention protocol for *H. influenzae* infection. Danish studies on the general epidemiology of *H. influenzae* are scarce, and a description of the serotype distribution and the related molecular resistance in Denmark since the introduction of the Hib vaccine is lacking.

In this study, we analyzed and described the epidemiology (serotype, MLST and antibiotic susceptibility including molecular resistance gene characteristics) of *H. influenzae* identified in invasive specimens in Denmark during the period 2014–2022, and investigating the possible impact/interference of COVID-19 with the epidemiology of invasive *H. influenzae* infections.

## 2 Materials and methods

### 2.1 Clinical isolates

Surveillance of invasive Hib infections in Denmark has since October 2007 included mandatory submission of isolates to the Neisseria and Streptococcus Reference Laboratory (NSR) at Statens Serum Institut (SSI) following an executive order (BEK nr 1102 af 20/09/2007), and although the surveillance specifies only Hib, the reference laboratory at SSI receives the majority of invasive *H. influenzae* isolates from all Danish regional laboratories of clinical microbiology (Slotved et al., 2022). With the exception of phenotypic ciprofloxacin data, all phenotypic susceptibility data pertaining to the *H. influenzae* cases were obtained from the MiBa database (Voldstedlund et al., 2014). These results are presented without specific breakpoints but rely on the individual interpretations made by microbiology departments following routine antimicrobial susceptibility testing, based on the EUCAST guideline (EUCAST guidelines).^4^

All isolates were collected from invasive cases in Denmark, defined as the presence of *H. influenzae* in a patient with a positive culture result from cerebrospinal fluid, blood, or other normally sterile sites. Serotype and sample site information for all clinical *H. influenzae* isolates between 2014 and 2022 was obtained from the Danish laboratory surveillance system at the Neisseria and Streptococcus Reference Laboratory.

### 2.2 Whole-genome sequencing and assembly

WGS was performed as previously described (Fuursted et al., 2016; Slotved et al., 2021). Briefly, genomic DNA was extracted using a DNeasy Blood & Tissue Kit (QIAGEN, Hilden, Germany) and fragment libraries were constructed using a Nextera XT DNA Library Preparation Kit (Illumina, US) followed by 150 or 250 bp paired-end sequencing on either the MiSeq or NextSeq 550 platform (Illumina, land), respectively, according to the manufacturer’s instructions. The paired-end data were *de novo* assembled using the SKESA assembler (SKESA v.2.2) (Souvorov et al., 2018).

The genomic sequence data for the 666 clinical isolates have been deposited at the European Nucleotide Archive (ENA) under Bioproject no. PRJEB56415 and project no. PRJEB67907.

### 2.3 Species identification and serotyping of *Haemophilus influenzae* isolates

The identification of *H. influenzae* was performed as previously described (Fuursted et al., 2016; Slotved et al., 2022). Briefly, species identification performed by matrix-assisted laser desorption/ionization time-of-flight mass spectrometry (MALDI-TOF MS) (Bruker Daltonics; Compass 1.4, version 3.4, build 3.4.76.0) was performed on strains transferred directly from bacterial colonies. Species identification was based on the standard MALDI-TOF score value [Biotyper database version MBT 6903 MSP Library (#1829023)] and confirmed with whole-genome sequencing (WGS) data using KmerFinder.^5^

All isolates were serotyped *in silico* (Watts and Holt, 2019) with default parameters, using the *in silico* program “hicap” version hicap 1.0.3: https://anaconda.org/bioconda/hicap, accessed 30-10-2023.

### 2.4 Antimicrobial resistance

Data on 730 *H. influenzae* isolates were available for both genotypic and phenotypic resistance analysis and comparison. Molecular analysis of beta-lactam resistance was done by blasting for beta-lactamase gene^6^ and if positive was termed genetic beta-lactamase-positive ampicillin-resistant (gBLPAS). Moreover, they were further analyzed and characterized by PBP3 substitutions (PBP group; Skaare et al., 2014) including *ftsI* allele typing (Giufrè et al., 2023).

Isolates with no PBP3 substitutions at amino acid position 517/526 and no detectable beta-lactamase genes were termed genetic beta-lactamase-negative ampicillin-susceptible (gBLNAS). Isolates with the PBP3 substitutions R517H or N526K were termed genetic beta-lactamase-negative ampicillin-resistant (gBLNAR). Isolates possessing both a beta-lactamase and PBP3 substitutions were termed genetic beta-lactamase-positive amoxicillin clavulanate resistant (gBLPACR).

A PBP3 substitution system has been introduced and refined (Ubukata, 2003; Dabernat and Delmas, 2012) to allow a PBP3 group typing system (Skaare et al., 2014). Determination of the *ftsI* allele was done by analyzing a 621-bp gene fragment corresponding to nucleotides 977–1,597 of the *ftsI* gene through the PubMLST database https://pubmlst.org/bigsdb?db=pubmlst_hinfluenzae_seqdef&page=sequenceQuery (Deghmane et al., 2019).

The genetic beta-lactam resistance mechanism was evaluated and compared with the phenotypic beta-lactam antibiogram extracted from the MiBa database. Detection of the quinolone resistance-determining region (QRDR) genes (*gyrA, gyrB, parC*, and *parE*), plasmid-mediated quinolone resistance genes (*qnrA1, qnrB6, qnrD*, and *qnrS*) was using BLASTN against *H. influenzae* isolate Rd. KW20.

Additional acquired antimicrobial resistance genes listed in the ResFinder database,^7^ such as aminoglycosides, tetracyclines, and others, were screened for their presence using ResFinder in our internal WGS setup.

### 2.5 Multilocus sequence typing (MLST)

MLST was performed by uploading the assembled genomes to the PubMLST database^8^ to identify sequence types (ST) and corresponding clonal complex (CC) for all isolates. MLST data from 2014–2021 has been presented in a previous study (Slotved et al., 2022). MLST data for received isolates for 2022 has been added.

### 2.6 Data analyses

Data were analyzed using Graph Pad Prism version 9.3.1 (GraphPad Software) for descriptive statistical analysis and confidence intervals (95% CI).

For calculation of all incidence data in this manuscript, we obtained population data on populations (per 100,000) for both specific age groups and total population from the Statistics Denmark homepage.^9^

Denmark is a geographically small country with the overall same weather and temperature conditions (costal conditions) for the entire country. Data on the temperature per month in Denmark is therefore representative for the entire country and was retrieved from our national weather service Danmarks Meteorologiske Institut (DMI).^10^

### 2.7 Ethical considerations

The data and samples from patients were collected routinely for national surveillance purposes, therefore no ethical approval or informed consent from patients or guardians were required. The study was approved by the Danish Data Protection Agency (record number 2007-41-0229). For further details on SSI’s permission to present and publish epidemiological data, see https://en.ssi.dk/research, accessed 30–06–2022, https://en.ssi.dk/about-us, accessed 30–06–2022. All presented data were anonymized.

## 3 Results

### 3.1 Epidemiology

Data on 1,007 invasive *H. influenzae* cases obtain between 2014 and 2022 of which we received 752 (74.7%) isolates for serotyping. For the remaining 255 cases were a viable isolate not available, and the isolates were marked as not typed using the abbreviation (NT).

**Figure 1:**
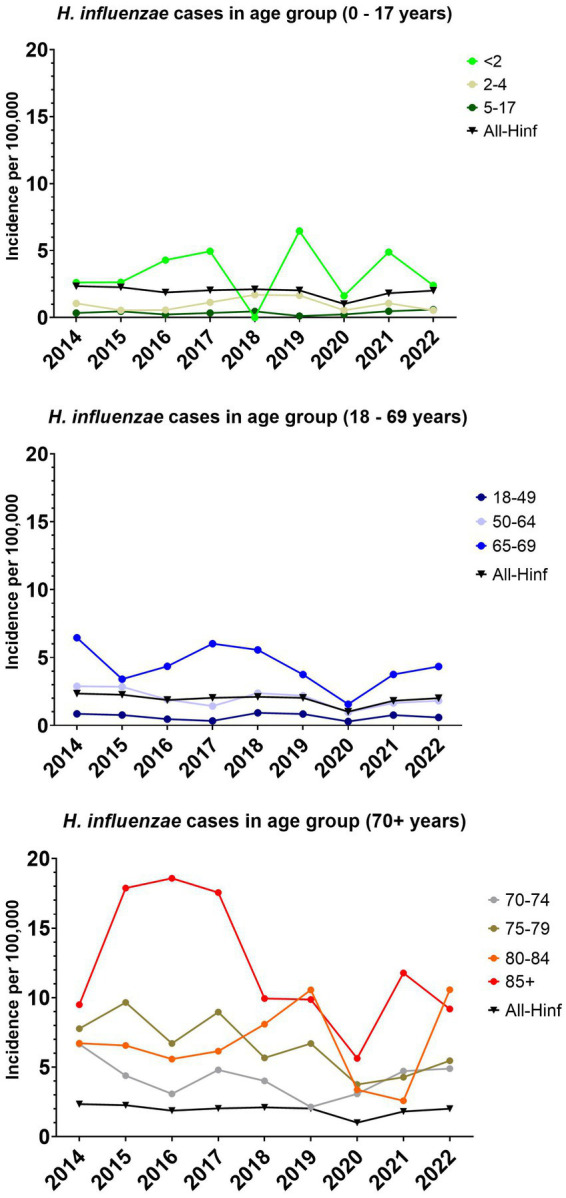
The incidence per 100,000 of invasive *Haemophilus influenzae* cases for all age groups and specific age groups.

For the entire population the overall median incidence of *H. influenzae* for the eight year period was 2.0 cases per 100,000 (CI 95% 1.8–2.3), with the lowest incidence in 2020 of 1.0 cases per 100,000 and the highest incidence of 2.3 cases per 100,000 in 2014 (Figure 1).

The majority were bacteremia cases (88.6%), 7.0% were meningitis cases, 3% pleura fluid, and the remaining 1.5% isolates came from various sites (synovial fluid, lung and epiglottis). For additional details on the description of the reported *H. influenzae* cases, see Table 1.

**Table 1:** Characteristic of the invasive *Haemophilus influenzae* isolates received at Statens Serum Institut from 2014 to 2022.

Invasive *H. influenzae* isolates from 2014–2022	1,007
Sex (female)	538 (53.1%)
Isolate information	
Blood	892 (88.6%)
Cerebrospinal fluid	70 (7.0%)
Pleural fluid	30 (3.0%)
Other sterile sites	15 (1.5%)
Age (Years) all patients with *H. influenzae* infection	
Median	68.0
Age Interquartile range	53.0–78.0
Age range	0–99

**Figure 2:**
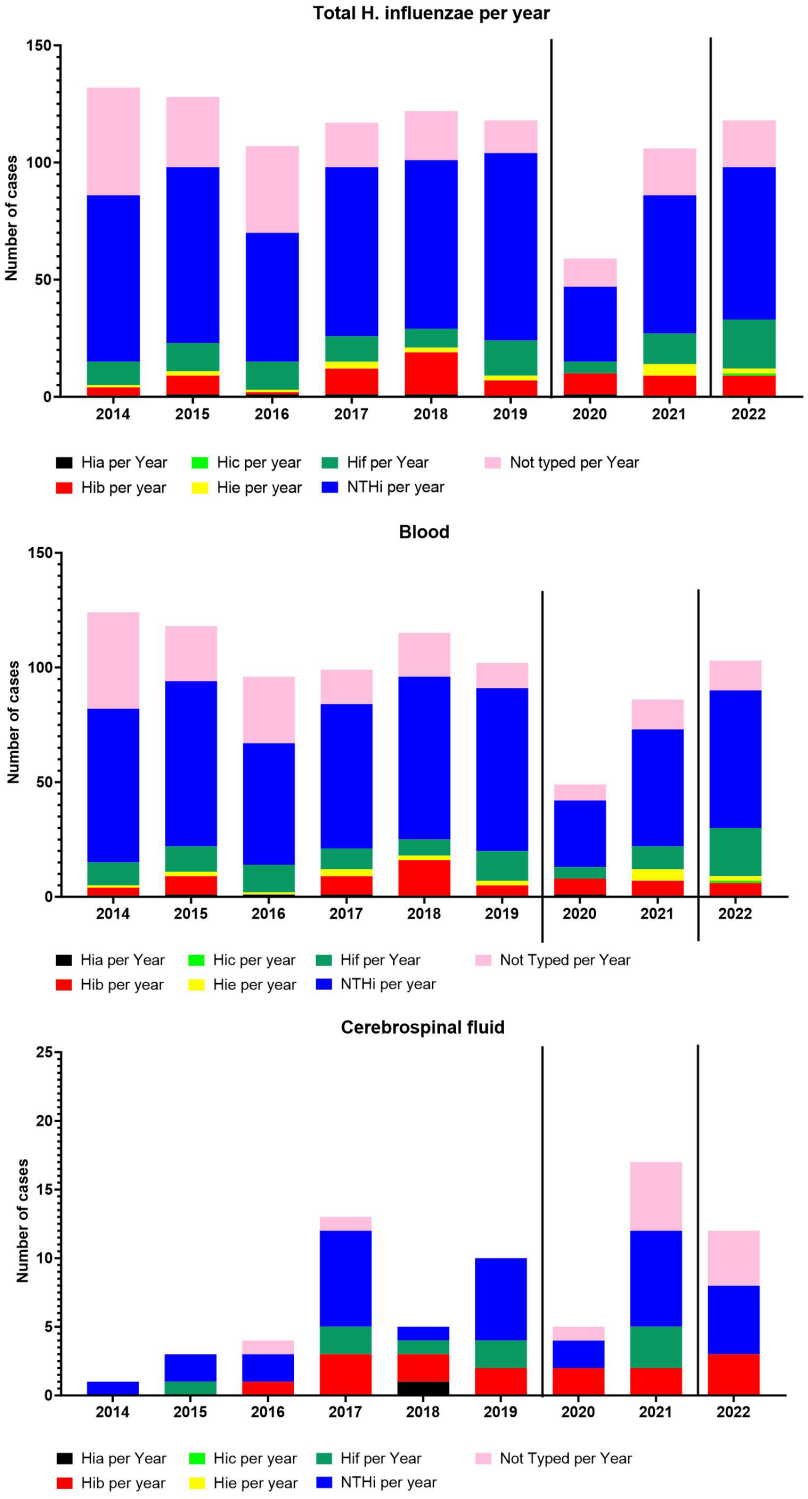
The number of total cases per year and serotype-specific cases per year. The number of cases from blood per year and serotype-specific cases per year. The number of cases from cerebrospinal fluid per year and serotype-specific cases per year.

The overall number of cases per year and the incidence per 100,000 has not changed greatly between 2014–2022, except for 2020 (Figures 1, 2). The number of meningitis cases has increased since 2014, although the numbers still are low (Figure 2).

The median incidence for children below two years of age, was 2.6 (95% CI, 1.6–4.9), with a range of 6.5. The median incidence for the age groups 2–64 years of age showed a median incidence below 2 (Figure 1). With the age group 65 and older, an increase in incidence was observed, with the largest median incidence observed in the age group above 85 with 9.9 per 100,000 (CI 9.2–17.9).

### 3.2 Serotype distribution

The serotype distribution fluctuated over the years (Figure 2), however, no major change was observed except for 2020, when the COVID-19 restriction were introduced (Tinggaard et al., 2023). In 2021 and 2022, we observed a return to pre-COVID-19-restrictions numbers. The NTHi *H. influenzae* dominated, followed by serotype f and serotype b.

The median incidence of Hib infection across all age groups was found to be low, at 0.18 per 100,000 individuals (76 isolates over the entire study period) (CI 0.1–0.19). This observation is noteworthy given that Hib vaccination is an integral component of the childhood vaccination program in Denmark, boasting a vaccination coverage exceeding 96% for the third vaccination.^11^ Nevertheless, it is notable that the highest median incidence, reaching 0.8 per 100,000 (CI 0.0–1.6), was still observed among children.

For the dominating type NTHi a total of 581 isolates were detected with a median incidence of 1.2 per 100,000 (CI 0.10–1.3), while for Hif, the second most observed serotype, 107 isolates were detected with a median incidence of 0.21 per 100,000 (CI 0.13–0.26). Only five cases of Hia were detected in the time period, with the most recent case dating back to 2020. For Hic only one case was detected in the period (2022), and for Hie 18 cases were detected during the period.

### 3.3 MLST type

The observed MLST types for the period 2014–2022 were generally differentiated into ST types related to capsular isolates and ST types only observed among the NTHi isolates, except for the ST103 (CC11) clone, which was observed among both Hic isolates and NTHi isolates. No other ST types were identified in either the capsular or NTHi isolates. For details, please refer to Supplementary Tables S1, S2.

### 3.4 COVID-19

In March 2020, preventive interventions to control the spread of COVID-19 were announced and implemented in Denmark in the following days (Emborg et al., 2021). The impact of the COVID-19 restrictions could be observed in 2020, with a significant reduction in the incidence of invasive *H. influenzae* infections (Figure 2). However, in 2021, a return to pre-COVID-19 levels of overall incidence of invasive *H. influenze* was observed (Figure 2). The individual serotypes of invasive *H. influenzae* also demonstrated a reduction in incidence in 2020, except for Hib, which appeared to be unaffected by the introduced restrictions (Figure 2).

### 3.5 Outdoor temperature effect

**Figure 3:**
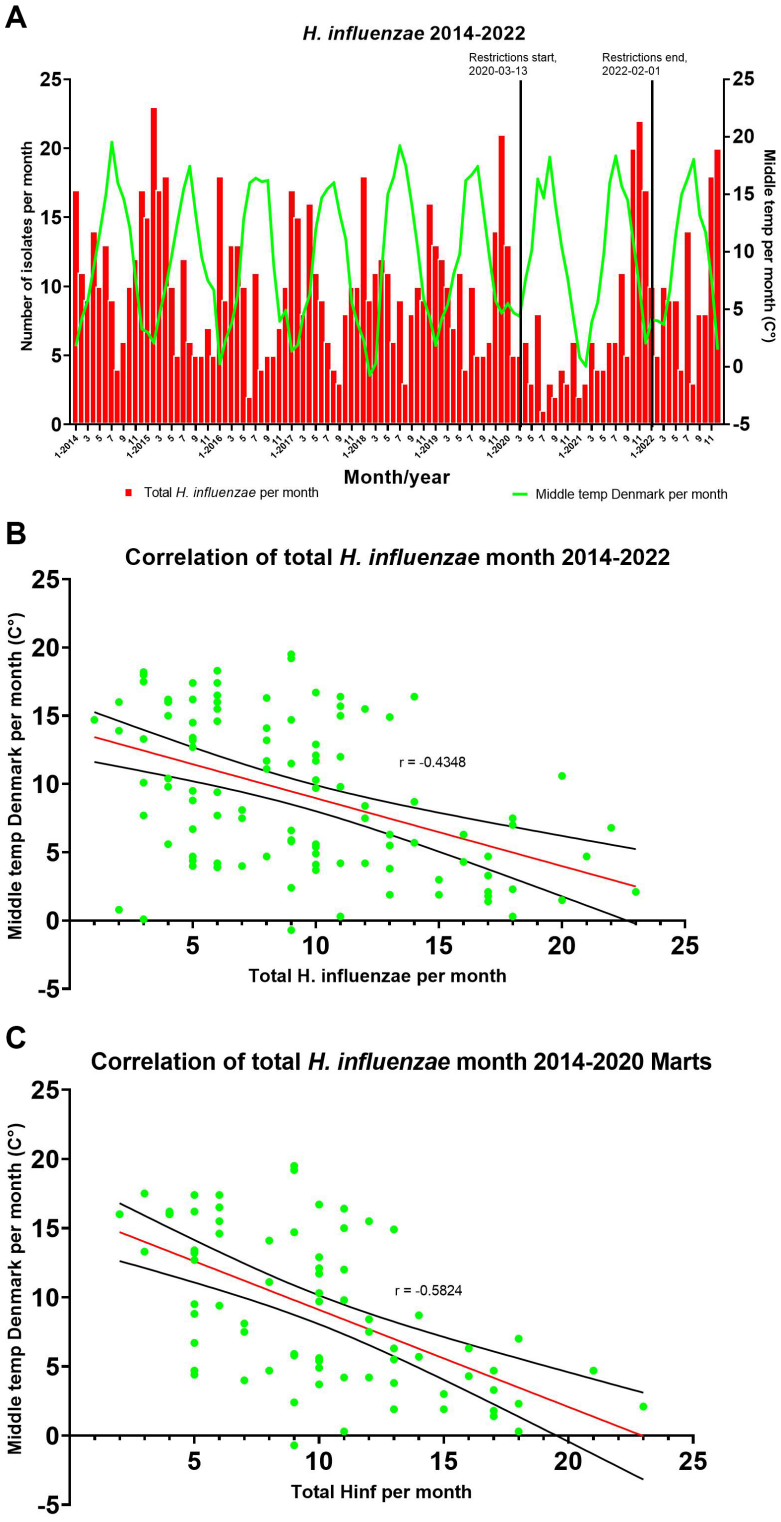
**(A)** Comparing seasons/temperature with the number of *H. influenzae* cases per month. **(B,C)** A simple linear regression has been added with a 95% confidence interval (CI).

Comparing the monthly total number of invasive cases of *H. influenzae* between 2014 and 2022 with the corresponding average outdoor temperature in Denmark demonstrated a significant negative correlation with a Spearman’s rank correlation coefficient of *r* = −0.43 (95% CI, −0.58 to −0.26) and a *p* value of <0.0001 (Figure 3).

After excluding data from March 2020 and onwards due to the impact of COVID-19 restrictions, there was an increase in the strength of the negative correlation between the monthly total number of *H. influenzae* from 2014 to March 2020 and the middle temperature in Denmark, with a Spearman’s rank correlation coefficient of *r* = −0.58 (95% CI, −0.72 to −0.40) and a *p* value of <0.0001 (Figure 3).

### 3.6 Antibiotic resistance

**Figure 4:**
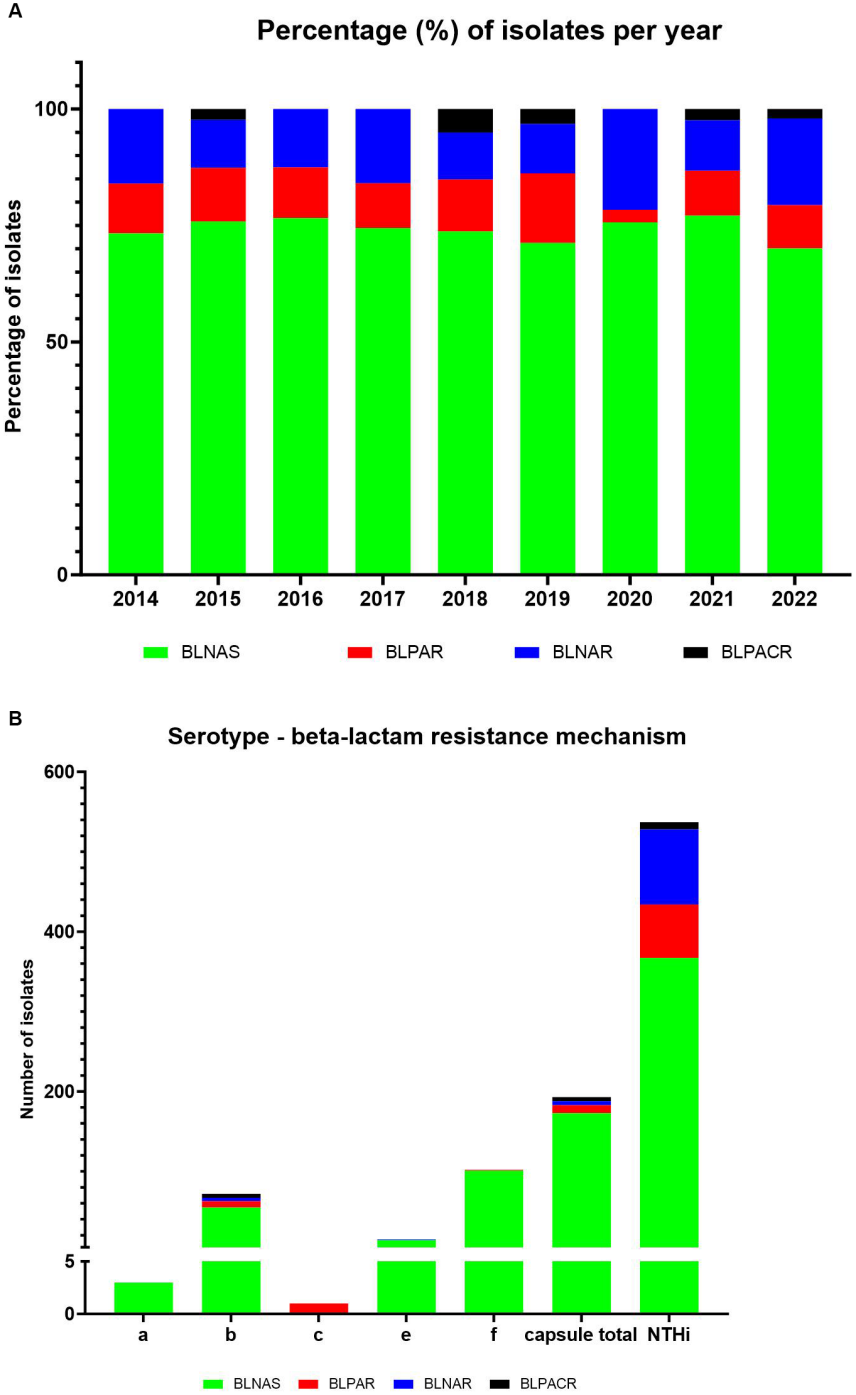
Distribution of genetic beta-lactam resistance mechanism in 730 sequenced *H. influenzae* isolates during the period 2014–2022. **(A)** Show percentage of isolates per year, while **(B)** show the serotype distribution.

The distribution of beta-lactam resistance mechanism during the 2014–2022 period can be seen in Figure 4 (for detailed numbers see Supplementary Table S3). An overall level of beta-lactam resistance of 26.3% was observed, divided into 10.6% gBLPAR, 13.6% gBLNAR, and 2.1% gBLPACR. Moreover, we observed more beta-lactam resistance in NTHi strains (31.7%) vs. capsular strains (10.4%) (Figure 4). The distribution of beta-lactam resistance mechanism between capsular and NTH strains revealed that gBLNAR and gBLPAR were primarily observed in NTH strains at 95 and 87%, respectively. Distribution of gBLNAS and gBLPACR were comparable in NTH strains (68 and 64%).

The change in the distribution of beta-lactam resistance mechanism during the period was overall minor, however a decrease in the proportion of BLPAR and an increase of BLNAR in 2020 (the first year with COVID-19) was observed (Figure 4).

We did not observe any larger differences in beta-lactam resistance mechanism with respect to site of infection, submitting laboratory within the five different regions of Denmark, or gender.

Beta-lactamase enzymes were detected in 91 (12.5%) of the isolates with TEM-1B as the most frequently detected (*n* = 71) followed by TEM-234 (*n* = 7), TEM-104 (*n* = 2) and TEM-1C (*n* = 1) while no ROB-1 beta-lactamase was observed (data not shown).

*ftsI* allele analysis allowed the identification of 73 different alleles varying from one to 134 isolates per allele (Supplementary Table S4). Distribution of *ftsI* alleles were very close related to serotypes – *ftsI* allele 46 was only observed in Hia, allele 55 only in Hie and allele 6 only in Hif, allele 10 was dominating (62/72) in Hib, while NTHi isolates had heterogenous distributed *ftsI* alleles (Supplementary Table S5). A similar relatedness was observed regarding *ftsI* allele and MLST-CC. Here *ftsI* alleles 6 was only observed in MLST-CC124, allele 43 only in CC107, and allele 27 was predominant (54/61) in CC11. The remaining alleles were scattered throughout the MLST-CC (Supplementary Table S6). *ftsI* allele 4, 6, 10, and 29 were exclusive observed in isolates with no PBP3 mutations belonging to gBLNAS and gBLPAR. PBP3 group I was only co-occurring with *ftsI* allele 97, PBP3 group IIa contained only allele 43 and 20, PBP3 group IId contained only allele 5, and 214 while PBP3 group IIb, IIc and III-like were found with various *ftsI* allele variants (Supplementary Table S7). The distribution of PBP3 group based on alterations in the *ftsl* gene encoding PBP3 can be seen in Supplementary Table S4. PBP3 group IIb was observed in more than 50% of the gBLNAR isolates followed by group IIa while PBP3 group IIb and IIc were dominant in gBLPACR isolates. PBP group III-like mutations were rarely detected (3.4%) and PBP group III total absent.

The frequency of mutations in the *fisl* gene of PBP3 varied in relation to the beta-lactam resistance mechanism (Table 2; Supplementary Tables S7, S8). However, of interest, the mutation V547I, N569S, D350N, and A586S were seen in more than 60% of the BLNAS isolates (Table 2).

**Table 2:** Mutations in the *ftsl* gene of PBP3 in relation to genetic beta-lactam resistance mechanism in 730 invasive *H. influenzae* isolates.

Mutations in *fisl* gene of PBP3	Reference	gBLNAS (%)	gBLPAR (%)	gBLNAR (%)	gBLPACR (%)	Total (%)
None		306 (85.2)	53 (14.8)	0	0	359 (47.7)
V547I	Barbosa et al. (2011) and Giufrè et al. (2023)	218 (69.4)	16 (5.1)	67 (21.3)	13 (4.1)	314 (41.8)
N569S	Barbosa et al. (2011) and Giufrè et al. (2023)	179 (69.9)	2 (0.8)	63 (24.6)	12 (4.6)	256 (34.0)
D350N	Barbosa et al. (2011) and Giufrè et al. (2023)	150 (62.8)	0	75 (31.4)	12 (5.0)	239 (31.8)
A586S	Tristram et al. (2007) and Cherkaoui et al. (2018)	120 (58.5)	10 (4.9)	63 (30.7)	12 (5.9)	205 (27.3)
N526K	Barbosa et al. (2011) and Giufrè et al. (2023)	0	0	87 (88.8)	11 (11.2)	98 (13.0)
A502T/V	Barbosa et al. (2011) and Giufrè et al. (2023)	2 (2.5)	0	66 (82.5)	12 (12.5)	80 (10.6)
M377I	Barbosa et al. (2011) and Giufrè et al. (2023)	0	0	52 (88.1)	7 (11.9)	59 (7.8)
A530S	Barbosa et al. (2011) and Giufrè et al. (2023)	0	0	21 (95.5)	1 (4.5)	22 (2.9)
R517H	Barbosa et al. (2011) and Giufrè et al. (2023)	0	0	11 (83.3)	1 (16.7)	12 (1.6)
A437S	Barbosa et al. (2011) and Giufrè et al. (2023)	10 (83.3)	0	2 (16.7)	0	12 (1.6)
S357N	Barbosa et al. (2011) and Giufrè et al. (2023)	0	0	4 (40.0)	6 (60.0)	10 (1.3)
I449V	Barbosa et al. (2011) and Giufrè et al. (2023)	0	0	6 (100)	0	6 (0.8)
S311P	Bae et al. (2010)	4 (100)	0	0	0	4 (0.5)
T532S	Barbosa et al. (2011) and Giufrè et al. (2023)	0	0	3 (75.0)	1 (25.0)	4 (0.5)
S385T	Barbosa et al. (2011) and Giufrè et al. (2023)	0	0	3 (75.0)	1 (25.0)	4 (0.5)
T443A	García-Cobos et al. (2007)	0	0	1 (100)	0	1 (0.1)

Underlined mutations N526K and R517H are BLNAR-defining mutations. See Supplementary Table S8 for amino acid substitutions in different PBP3 subtypes.

We also compared the recorded beta-lactam antibiogram from the MiBa database (Voldstedlund et al., 2014) with the determined genetic beta-lactam resistance mechanism (Table 3). A very high concordance between genotypic and phenotypic beta-lactam resistance was observed at 98.2%. We found a 100% concordance between the phenotypic and genotypic beta-lactam resistance regarding beta-lactamase-producing isolates (gBLPAR and gBLPACR) and in isolates with no detectable resistance genes (gBLNAS). In contrast 12 (13.1%) isolates of the gBLNAR isolates were phenotypically registered as ampicillin/amoxicillin susceptible. These were all NTHi, except one Hib, and were equally distributed regarding geographical origin, PBP3 group and *ftsI* allele.

**Table 3:** Concordance between the phenotypic beta-lactam antibiogram and genetic beta-lactam resistance mechanism in 730 invasive *H. influenzae* isolates.

Beta-lactam resistance mechanism (%)				
Penicillin/ampicillin antibiogram	gBLNAS	gBLPAR	gBLNAR	gBLPACR
Susceptible	539 (100)	0	13 (13)	0
Resistant	0	78 (100)	86 (87)	14 (100)
Total	539	78	99	14

Five ciprofloxacin resistant isolates were detected with various well-known mutation observed; all were mono-resistant to ciprofloxacin except for one isolate (gBLPACR) and number of mutations correlated with ciprofloxacin MIC (Table 4).

**Table 4:** Ciprofloxacin resistance versus mutations.

Isolate number	Reference	2021–0088	2019–2087	2018–2054	2017–1922	2014–1,353	Hinf-rf-KW20 (L42023)-ref
Serotype		E	NTHi^1^	NTHi	NTHi	NTHi	F
MLST		18	422	1,524	139	1,521	1,621
gyrA							
S84L	Chen et al. (2018), Fuursted et al. (2016), and Shoji et al. (2014)	S	L	L	L	L	S
D88N	Fuursted et al. (2016) and Shoji et al. (2014)	D	D	G	D	N	D
M121L	Shoji et al. (2014)	M	M	M	M	M	M
E142L	Shoji et al. (2014)	E	E	E	E	E	E
gyrB							
A400V	Shoji et al. (2014)	A	A	A	V	A	A
parC							
K20R	Fuursted et al. (2016)	K	K	R	R	R	K
G82A/C	Fuursted et al. (2016) and Shoji et al. (2014)	G	G	G	G	G	G
S84I/R	Chen et al. (2018), Fuursted et al. (2016), and Shoji et al. (2014)	S	R	I	I	I	S
Q/E88L	Fuursted et al. (2016) and Shoji et al. (2014)	E	E	E	E	E	E
S133A	Shoji et al. (2014)	A	S	S	S	A	S
N138S	Shoji et al. (2014)	S	N	S	S	S	N
D/T356A	Fuursted et al. (2016)	T	D	D	A	A	D
M481I	Fuursted et al. (2016)	M	M	M	M	I	M
parE							
E151K	Fuursted et al. (2016)	E	E	E	E	K	E
R381C	Shoji et al. (2014)	R	R	R	R	R	R
D420N	Fuursted et al. (2016)	D	D	N	D	N	D
V466M	Shoji et al. (2014)	V	M	V	V	V	V
S599A	Fuursted et al. (2016)	S	S	S	A	A	S
Number of mutations		2	3	6	7	12	0
Ciprofloxacin MIC (mg/L)		0.125	1	1	>32	>32	0.03
Other resistance traits		None	BLPACR	None	None	None	None

NTHi, non-typeable *H. influenzae*.

We detected 55 other (non-beta-lactam, non-quinolone) resistance genes in the 730 *H. influenzae* isolates, primarily aminoglycoside-modifying enzymes (39 isolates) but also toward tetracycline (eight isolates), sulfonamides (six isolates) and macrolides (two isolates) (Supplementary Table S9).

## 4 Discussion

This study presents an overview of Danish invasive clinical *H. influenzae* isolates and their antimicrobial resistance from 2014 to 2022. The introduction of the Hib vaccine in the 1990s led to a significant reduction in Hib-caused infections worldwide. However, this reduction was followed by serotype replacement, primarily affecting age groups other than children (Watt et al., 2009; Jalalvand and Riesbeck, 2018; McElligott et al., 2020; Nørskov-Lauritsen et al., 2021; Tønnessen et al., 2022). A previous study in Denmark, which included data from 2000 to 2008, reported an incidence rate of around 1.5 per 100,000 population for the Danish population (Laupland et al., 2011). Another Danish study estimated the prevalence of *H. influenzae* in clinical samples on January 10th, 2018, to be around 1.78 per 100,000 person-days (all samples) (Nørskov-Lauritsen et al., 2021). In this study, a similar median incidence rate of 2.0 per 100,000 was observed (Figure 1). In studies from several different countries, the incidence of Hia in children has appeared to increase, with a number of reports of invasive Hia disease coming from Alaska, northern Canada, as well as New Mexico and Utah (Crandall et al., 2020). In Norway, an increase in the incidence of Hia from 2017–2021 was also observed, despite the COVID-19 restrictions (Tønnessen et al., 2022). However, this does not seem to be the situation in Denmark, where only five cases of Hia were detected in the study period. Similarly, in Ireland, Hia was found to be a rare cause of invasive disease (McElligott et al., 2020). In Utah, USA, a predominance of Hia ST62 strains was found (Crandall et al., 2020), which was not observed in this study. We found that all five cases of Hia in Denmark belonged to the CC-23 (Slotved et al., 2022). NTHi has been reported as the dominant disease-causing serotype in several countries (McElligott et al., 2020), which is also the case in this study, where NTHi were found to be predominant (Figure 2).

In our previous study (Slotved et al., 2022), did we observe that the STs/CC observed in the capsular isolates were not found among the NTHi isolates, and vice versa. However, in 2022, we found an Hic isolate belonging to the ST type 103/CC11. This clone was also identified among the NTHi isolates. This observation supports earlier findings (Potts et al., 2019), highlighting that MLST cannot reliably identify specific capsular serotypes.

The implementation of preventive measures to combat COVID-19 resulted in a noticeable decline in invasive *H. influenzae* cases in Denmark and various other countries (Brueggemann et al., 2021; Shaw et al., 2023). Nevertheless, the decrease proved to be temporary in Denmark as it reverted back to pre-COVID-19 levels by 2021 (Figure 2).

Studies have shown that invasive *H. influenzae* infections follow a yearly seasonal pattern, with a higher incidence observed during winter as compared to the rest of the year (Paireau et al., 2016; Wilkinson et al., 2017). However, these studies did not investigate the correlation between infection incidence and temperature. We therefore propose a hypothesis that suggests it is not solely the seasonal variation but rather the temperature that exhibits a correlation with the number of *H. influenzae* cases in Denmark. This hypothesis gains particular relevance in light of the climate changes observed worldwide, where seasons may not be as consistent as a parameter, but temperature variations are anticipated to play a more significant role in influencing infection rates. Comparing the monthly mean outdoor temperature in Denmark with the monthly number of *H. influenzae* cases showed a seasonal variation, with most cases occurring in the colder winter season (Figure 3). Additionally, comparing the monthly mean temperature for Denmark showed that the cases followed the temperature level and that the temperature was negatively correlated with the incidence (Figure 3).

The emergence and spread of antibiotic resistance in *H. influenzae* strains present a notable challenge in effectively addressing invasive infections (Tønnessen et al., 2022; Giufrè et al., 2023). This study aimed to analyze the molecular characteristics of various antimicrobial resistance traits among the collected *H. influenzae* isolates.

Our findings revealed an overall beta-lactam resistance rate of 26.3% within the three classic distinct resistance mechanisms (gBLPAR, gBLNAR, and gBLPACR). These results are consistent with previous reports highlighting the widespread occurrence of these resistance mechanisms (Tønnessen et al., 2022; Giufrè et al., 2023). Notably, we observed a decrease in the frequency of gBLPAR isolates and an increase in gBLNAR isolates in 2020, coinciding with the first year of COVID-19. This finding suggests a potential impact of the pandemic and associated interventions on the distribution of beta-lactam resistance mechanisms. Further studies are warranted to explore the underlying factors contributing to this observed shift.

We did not observe any greater differences in the distribution of beta-lactam resistance mechanisms based on the site of infection, submitting laboratory within the five different regions of Denmark, or gender. However, we observed a higher prevalence of beta-lactam resistance in NTHi strains (31.7%) compared to capsular strains (10.4%), which were observed primarily and comparably in gBLNAR (95%) and gBLPAR (87%) isolates. This is an interesting observation because BLPAR is reported to spread via conjugal plasmid transfer and gBLNAR by de-novo mutation or inter-strain transformation (Skaare et al., 2014; Tønnessen et al., 2022). We have no explanation why there were no difference, especial as we observed less variation in ftsI allele in capsular strains – consistent with them probably being less amenable to natural transformation. However, this highlights the importance of monitoring and understanding the dynamics of resistance in different *H. influenzae* populations, as it can have implications for treatment strategies and vaccine development.

Beta-lactamase enzymes play a crucial role in conferring resistance to beta-lactam antibiotics. Among the isolates tested, we detected genes encoding beta-lactamase enzymes in 12.5% of the isolates, with TEM-1B being the most frequently detected variant. This finding is consistent with previous studies demonstrating the predominance of TEM-type beta-lactamases in *H. influenzae* (Tønnessen et al., 2022; Giufrè et al., 2023).

Additionally, we investigated the genetic distribution of penicillin-binding protein 3 (PBP3) groups based on alterations in the *ftsI* gene. PBP3 group IIb was observed in more than 50% of gBLNAR isolates, followed by group Iia. In gBLPACR isolates, PBP3 groups IIb and IIc were more prevalent, while PBP group III-like alterations were rare. These findings indicate the importance of PBP3 alterations in mediating resistance to beta-lactam antibiotics and highlight the need for continuous surveillance of PBP3 variants.

Furthermore, we identified non-defining mutations in the *ftsI* gene associated with the beta-lactam resistance mechanism (Table 2; Supplementary Table S8). Notably, the mutations V547I, N569S, and D350N were present in over 60% of BLNAS isolates. These mutations have previously been reported to confer resistance to beta-lactam antibiotics and contribute to the gBLNAR phenotype (Barbosa et al., 2011; Giufrè et al., 2023).

The concordance between genotypic and phenotypic beta-lactam resistance was high (98.2%). The concordance between genotypic resistance mechanism and phenotypic beta-lactam resistance was 100% for beta-lactamase-producing isolates (gBLPAR and gBLPACR) and gBLNAS isolates. However, a considerable proportion (13.1%) of gBLNAR isolates were phenotypically registered as ampicillin/amoxicillin susceptible, indicating potential challenges in accurately detecting and characterizing resistance in these isolates.

Other non-beta-lactam resistance traits were detected in 60 isolates. Fifty-five isolates possessed various resistance genes, primarily aminoglycoside-modifying enzymes but also resistance genes toward tetracycline, sulfonamides, and macrolides. Five isolates were ciprofloxacin resistant with various well-known mutations observed and the number of mutations correlated with ciprofloxacin MIC. Of interest, one (blood) isolate from Region of Northern Denmark (#2014-1353), a high-level ciprofloxacin-monoresistant *H. influenzae* isolate (ST 1521) appeared to be part of the clonal occurrence described in Region of Southern Denmark of sputum, ear and eye samples (Fuursted et al., 2016). We have no information about a possible epidemiological link between these patients living in the two distinct geographical regions.

A limitation of this study is that only Hib isolates are mandatory for submission to the Danish regional laboratories of clinical microbiology. This may lead to an underestimation of the number of cases of all other serotypes compared to Hib cases. However, it should be noted that serotyping of *H. influenzae* is not a routine part of the testing protocol in these laboratories, and all laboratories submit isolates of all types. Therefore, we believe that the 25% of cases in which we do not receive an isolate are representative of all serotypes equally.

Approximately 75% of isolates from all *H. influenzae* cases registered in the MiBa database (Voldstedlund et al., 2014), were received at SSI, resulting in a lack of serotyping data for the remaining 25% of the cases. However, we believe that the 25% of cases not received are random and exhibit limited bias toward a specific serotype.

Another limitation is, that with the exception of phenotypic ciprofloxacin data, all phenotypic susceptibility data pertaining to *H. influenzae* cases were sourced from the MiBa database. The results do not include specific zone diameters/MIC value, but only report the interpretative category (SIR). All laboratories adhere to the EUCAST guidelines and EUCAST breakpoints. Moreover, laboratories do not report the beta-lactam resistance mechanism inferred from EUCAST’s algorithm screening flow chart.

The strength of this study is its nationwide scope, with representation from all departments of clinical microbiology in Denmark. This provides a comprehensive understanding of *H. influenzae* epidemiology and antibiotic resistance in Denmark.

In conclusion, the incidence of *H. influenzae* in Denmark remained relatively stable between 2014 and 2022, with an overall median incidence of 2.0 cases per 100,000. However, the implementation of COVID-19 preventive interventions in 2020 resulted in a substantial reduction in *H. influenzae* incidence, which returned to pre-COVID levels in 2021. The majority of cases were bacteremia cases, with the highest incidence observed in the age group 85 and above. NTHi was the most frequently detected serotype, followed by Hif and Hib. A significant negative correlation between the incidence of *H. influenzae* and temperature was observed. The study also revealed an overall genetic beta-lactam resistance rate of 26.3%, with high concordance between genotypic and phenotypic beta-lactam resistance.

## Data availability statement

The datasets presented in this study can be found in online repositories. The names of the repository/repositories and accession number(s) can be found in the article/Supplementary material.

## Ethics statement

SSI holds a general approval from the Danish Data Protection Agency (record number 2007-41-0229) (https://en.ssi.dk/research, https://en.ssi.dk/about-us). The studies were conducted in accordance with the local legislation and institutional requirements. Written informed consent for participation was not required from the participants or the participants’ legal guardians/next of kin in accordance with the national legislation and institutional requirements.

## Author contributions

H-CS: Conceptualization, Data curation, Formal analysis, Methodology, Writing – original draft. TJ: Data curation, Methodology, Writing – review & editing. MS: Data curation, Methodology, Writing – review & editing. TD: Data curation, Writing – review & editing. KF: Conceptualization, Data curation, Investigation, Writing – original draft.

## Footnotes

^1^https://statistik.ssi.dk//sygdomsdata#!/?vaccination=3&sex=3&landsdel=100&xaxis=Cohort&show=Graph&datatype=Vaccination, accessed 17-12-2021.

^2^https://www.ssi.dk/vaccinationer/boernevaccination/sygdomsforekomst-foer-og-efter-vaccination, accessed 29-11-2021.

^3^ https://www.danmap.org/reports

^4^ https://www.eucast.org/clinical_breakpoints

^5^ https://cge.cbs.dtu.dk/services/KmerFinder/

^6^ http://bldb.eu/

^7^https://cge.food.dtu.dk/services/ResFinder/, accessed 07-09-2023.

^8^https://pubmlst.org/organisms/haemophilus-influenzae, accessed 09-08-2023.

^9^https://www.dst.dk/en, accessed 21-04-2023.

^10^https://www.dmi.dk/, accessed 21-04-2023.

^11^https://statistik.ssi.dk/, accessed on 07-05-2023.
