# Supplementary material for: National Danish surveillance of invasive clinical Haemophilus influenzae isolates and their resistance profile
Source: Front Microbiol. 2023 Nov 22;14:1307261. doi: 10.3389/fmicb.2023.1307261 (PMC10702724; doi:10.3389/fmicb.2023.1307261)
Supplement: Supplementary file 1 [file Table_1.docx]

Article Title: National Danish surveillance of invasive clinical *Haemophilus influenzae* isolates and their resistance profile

Hans-Christian Slotved^1^*, Thor Bech Johannesen^1^, Marc Stegger^1^, Tine Dalby^2^, Kurt Fuursted^1^.

^1^Department of Bacteria, Parasites and Fungi, Statens Serum Institut, Copenhagen, Denmark.

^2^Department of Infectious Disease Epidemiology & Prevention, Statens Serum Institut, Copenhagen, Denmark.

*** Correspondence:**Hans-Christian Slotved, Department of Bacteria, Parasites and Fungi,

Bldg. 45/305, Artillerivej 5, DK-2300 Copenhagen S, Denmark.

Phone: +45 32688422, E-mail: [hcs@ssi.dk](mailto:hcs@ssi.dk)

Keywords: Denmark, epidemiology, resistance profile, *Haemophilus influenzae*.

# Reference

Slotved, H., Johannesen, T. B., Stegger, M., and Fuursted, K. (2022). Evaluation of molecular typing for national surveillance of invasive clinical Haemophilus influenzae isolates from Denmark. *Front. Microbiol.* 13, 1030242. doi: 10.3389/fmicb.2022.1030242.

Supplementary table 1. Updated data on the sequence type and clonal complex found among Danish *H. influenzae* isolates with a capsule (2014-2022) (Slotved et al., 2022).

| ST | Clonal complex | A | B | C | D | E | F | NTHi^b^ |
| --- | --- | --- | --- | --- | --- | --- | --- | --- |
| 56 | ST-23 complex | + |  |  |  |  |  | - |
| 2053 | ST-23 complex | + |  |  |  |  |  | - |
| 2057 | ST-23 complex | + |  |  |  |  |  | - |
| 6 | ST-6 complex |  | + |  |  |  |  | - |
| 92^a^ | ST-6 complex |  | + |  |  |  |  | - |
| 95 | ST-6 complex |  | + |  |  |  |  | - |
| 190 | ST-6 complex |  | + |  |  |  |  | - |
| 206 | ST-6 complex |  | + |  |  |  |  | - |
| 709 | ST-6 complex |  | + |  |  |  |  | - |
| 1448 | ST-6 complex |  | + |  |  |  |  | - |
| Novel | ST-6 complex |  | + |  |  |  |  | - |
| 9 | ST-7 complex |  |  | + |  |  |  | - |
| 103^a^* | ST-11 complex* |  |  | + |  |  |  | + |
| 47 | ST-10 complex |  |  |  | + |  |  | - |
| 18 | ST-18 complex |  |  |  |  | + |  | - |
| 122 | ST-18 complex |  |  |  |  | + |  | - |
| 386 | ST-18 complex |  |  |  |  | + |  | - |
| Novel | ST-18 complex |  |  |  |  | + |  | - |
| 124 | ST-124 complex |  |  |  |  |  | + | - |
| 598 | ST-124 complex |  |  |  |  |  | + | - |
| 1739 | ST-124 complex |  |  |  |  |  | + | - |

a: new ST types found in 2022.

b: Non-Typeable *H. influenzae* (NTHi).

*: Observed both as capsular and non-capsular.

Supplementary table 2. Updated data on the sequence type and clonal complex found among Danish Non-typeable *H. influenzae* (NTHi) isolates (2014-2022) (Slotved et al., 2022), * new ST types found in 2022.

| ST | Clonal complex | ST | Clonal  complex | ST | Clonal complex | ST | Clonal complex | ST | Clonal complex | ST | Clonal complex |
| --- | --- | --- | --- | --- | --- | --- | --- | --- | --- | --- | --- |
| 2 | ST-422 | 146 | None | 266 | ST-266 | 567 | ST-746 | 1041 | None | 1850 | ST-393 |
| 3 | ST-3 | 147 | ST-836 | 276 | ST-264 | 582 | ST-3 | 1054 | ST-266 | 1904 | ST1426 |
| 11 | ST-11* | 155 | ST-155 | 311 | None | 589 | ST-589 | 1069 | ST-183 | 2031 | ST-3 |
| 12 | ST-12 | 156 | ST-472 | 334 | None | 597 | ST-584 | 1076 | ST-107 | 2156 | None |
| 13 | ST-105 | 159 | ST-107 | 348 | ST-348 | 608 | ST513 | 1144 | None | 2332 | ST-836 |
| 14 | ST-3 | 160 | ST-487 | 349 | ST-321 | 634 | None | 1170 | ST-1836 | 2333 | None |
| 34 | ST-34 | 161 | ST-1477 | 351 | ST-199 | 652 | ST-652 | 1198 | ST-746 | 2519 | None |
| 41 | ST-41 | 165 | ST-165 | 367 | ST-3 | 653 | ST-3 | 1202 | ST-396 | Novel | ST-107 |
| 43 | None | 176 | ST-210 | 368 | ST-1836 | 690 | ST-264 | 1215 | None | Novel | ST-163 |
| 46 | ST-3 | 180 | ST-3 | 388 | ST-395 | 697 | ST-3 | 1218 | ST-107 | Novel | ST-199 |
| 57 | ST-57 | 183 | ST-183 | 389 | ST-389 | 804 | None | 1220 | ST-210 | Novel | ST-210 |
| 60^a^ | None | 187 | ST-584 | 393 | ST-389 | 835 | None | 1238 | ST-931 | Novel | ST-264 |
| 84 | ST-84 | 196 | None | 408 | ST-3 | 836 | ST-836 | 1379 | ST-584 | Novel | ST-266 |
| 85^a^ | ST-472 | 199 | ST-199 | 409 | ST-3 | 838 | ST-1426 | 1382 | None | Novel^a^ | ST-396 |
| 98 | ST-57 | 200 | None | 411 | ST-422 | 841 | None | 1401 | None | Novel | ST-422 |
| 103* | ST-11* | 201 | ST-107 | 422 | ST-422 | 914 | ST-139 | 1426 | ST-1426 | Novel | ST-425 |
| 105 | ST-105 | 203 | None | 425 | ST-425 | 925 | None | 1497 | ST-3 | Novel^a^ | ST-472 |
| 107 | ST-107 | 208 | ST-210 | 427 | ST-992 | 932 | None | 1521 | None | Novel | ST-519 |
| 113 | ST-1426 | 210 | ST-210 | 436 | ST-3 | 943 | None | 1524 | ST-3 | Novel | ST-584 |
| 134 | ST-11* | 241 | None | 472 | ST-472 | 946 | None | 1591 | ST-836 | Novel | ST-836 |
| 136 | ST-3 | 245 | ST-836 | 474 | ST-474 | 949 | None | 1714 | ST-519 | Novel | ST-1529 |
| 139 | ST-139 | 249 | ST-249 | 485 | ST-107 | 958 | ST-12 | 1727 | None | Novel | ST-1836 |
| 142 | ST-142 | 253 | ST-395 | 524 | ST-163 | 990 | ST-393 | 1773 | ST-107 | Novel | None |
| 143 | ST-3 | 262 | ST-262 | 531 | None | 995 | None | 1780 | None |  |  |
| 145 | ST-11* | 264 | ST264 | 556 | ST-395 | 1034 | ST-3 | 1834 | ST-931 |  |  |

a: new ST types found in 2022

*: Observed both as capsular and NTHi.

Supplementary table 3. Distribution of genetic beta-lactam resistance mechanism in 730 sequenced *H. influenzae* isolates during the period 2014-2022.

| **Year** | **Genetic beta-lactam resistance mechanism (%)** | | | | |
| --- | --- | --- | --- | --- | --- |
|  | gBLNAS* | gBLPAR | gBLNAR | gBLPACR | Total |
| 2014 | 55 (72.0) | 8 (11.0) | 12 (17.1) | 0 | 75 |
| 2015 | 66 (75.8) | 10 (11.0) | 9 (11.0) | 2 (2.2) | 87 |
| 2016 | 49 (76.6) | 7 (10.9) | 8 (12.5) | 0 | 64 |
| 2017 | 70 (73.5) | 9 (10.2) | 15 (16.3) | 0 | 94 |
| 2018 | 73 (73.7) | 11 (11.1) | 10 (9.1) | 5 (6.1) | 99 |
| 2019 | 67 (70.7) | 14 (15.2) | 10 (10.1) | 3 (4.0) | 94 |
| 2020 | 27 (75.7) | 2 (2.7) | 8 (21.6) | 0 | 37 |
| 2021 | 64 (77.4) | 8 (9.5) | 9 (10.7) | 2 (2.4) | 83 |
| 2022 | 68 (70.4) | 9 (9.2) | 18 (18.4) | 2 (2.0) | 97 |
| All year | 539 (73.7) | 78 (10.6) | 99 (13.6) | 14 (2.1) | 730 |

*gBLNAS = beta-lactamase negative ampicillin susceptible; gBLPAR = beta-lactamase positive ampicillin resistant,

gBLNAR = beta-lactamase negative ampicillin resistant; gBLPACR = beta-lactamase positive amoxicillin-clavulate resistant

Supplementary table 4. Frequency of the determined *ftsI* allele versus PBP3 group in 730 invasive *H. influenzae* isolates.

|  |  | **PBP3 group** | |  |  |  |  |  |
| --- | --- | --- | --- | --- | --- | --- | --- | --- |
| ***ftsI* allele** | **No mutation** | **I** | **IIa** | **IIb** | **IIc** | **IId** | **III like** | **Total** |
| 10 | 134 | 0 | 0 | 0 | 0 | 0 | 0 | 134 |
| 6 | 101 | 0 | 0 | 0 | 0 | 0 | 0 | 101 |
| 8 | 59 | 0 | 0 | 1 | 0 | 0 | 0 | 60 |
| 27 | 59 | 0 | 0 | 0 | 0 | 0 | 1 | 60 |
| 37 | 57 | 0 | 0 | 0 | 0 | 0 | 0 | 57 |
| 4 | 39 | 0 | 0 | 0 | 0 | 0 | 0 | 39 |
| 1 | 1 | 0 | 0 | 24 | 0 | 0 | 0 | 25 |
| 2 | 0 | 0 | 0 | 22 | 0 | 0 | 0 | 22 |
| 39 | 22 | 0 | 0 | 0 | 0 | 0 | 0 | 22 |
| 43 | 1 | 0 | 20 | 0 | 0 | 0 | 0 | 21 |
| 18 | 18 | 0 | 0 | 0 | 0 | 0 | 0 | 18 |
| 55 | 14 | 0 | 0 | 1 | 0 | 0 | 0 | 15 |
| 34 | 13 | 0 | 0 | 0 | 0 | 0 | 0 | 13 |
| 29 | 9 | 0 | 0 | 0 | 0 | 0 | 0 | 9 |
| 50 | 8 | 0 | 0 | 0 | 0 | 0 | 0 | 8 |
| 63 | 8 | 0 | 0 | 0 | 0 | 0 | 0 | 8 |
| 15 | 7 | 0 | 0 | 0 | 0 | 0 | 0 | 7 |
| 116 | 7 | 0 | 0 | 0 | 0 | 0 | 0 | 7 |
| Closes match: 342 | 0 | 0 | 0 | 0 | 6 | 0 | 0 | 6 |
| 12 | 5 | 0 | 0 | 0 | 0 | 0 | 0 | 5 |
| 104 | 5 | 0 | 0 | 0 | 0 | 0 | 0 | 5 |
| 5 | 0 | 0 | 0 | 0 | 0 | 4 | 0 | 4 |
| 23 | 0 | 0 | 0 | 4 | 0 | 0 | 0 | 4 |
| 48 | 0 | 0 | 0 | 4 | 0 | 0 | 0 | 4 |
| 53 | 4 | 0 | 0 | 0 | 0 | 0 | 0 | 4 |
| 97 | 0 | 4 | 0 | 0 | 0 | 0 | 0 | 4 |
| 126 | 4 | 0 | 0 | 0 | 0 | 0 | 0 | 4 |
| 135 | 4 | 0 | 0 | 0 | 0 | 0 | 0 | 4 |
| 46 | 3 | 0 | 0 | 0 | 0 | 0 | 0 | 3 |
| 106 | 3 | 0 | 0 | 0 | 0 | 0 | 0 | 3 |
| Closes match: 15 | 3 | 0 | 0 | 0 | 0 | 0 | 0 | 3 |
| 9 | 0 | 0 | 0 | 2 | 0 | 0 | 0 | 2 |
| 20 | 0 | 0 | 2 | 0 | 0 | 0 | 0 | 2 |
| 21 | 0 | 0 | 0 | 0 | 2 | 0 | 0 | 2 |
| 32 | 0 | 0 | 0 | 0 | 0 | 0 | 2 | 2 |
| 70 | 2 | 0 | 0 | 0 | 0 | 0 | 0 | 2 |
| 132 | 2 | 0 | 0 | 0 | 0 | 0 | 0 | 2 |
| 137 | 2 | 0 | 0 | 0 | 0 | 0 | 0 | 2 |
| 156 | 2 | 0 | 0 | 0 | 0 | 0 | 0 | 2 |
| 214 | 0 | 0 | 0 | 0 | 0 | 2 | 0 | 2 |
| 13 | 0 | 0 | 0 | 0 | 1 | 0 | 0 | 1 |
| 17 | 0 | 0 | 0 | 0 | 1 | 0 | 0 | 1 |
| 31 | 1 | 0 | 0 | 0 | 0 | 0 | 0 | 1 |
| 33 | 0 | 0 | 0 | 0 | 0 | 0 | 1 | 1 |
| 35 | 0 | 0 | 0 | 1 | 0 | 0 | 0 | 1 |
| 38 | 0 | 0 | 0 | 0 | 1 | 0 | 0 | 1 |
| 52 | 0 | 1 | 0 | 0 | 0 | 0 | 0 | 1 |
| 56 | 1 | 0 | 0 | 0 | 0 | 0 | 0 | 1 |
| 75 | 0 | 0 | 0 | 1 | 0 | 0 | 0 | 1 |
| 111 | 1 | 0 | 0 | 0 | 0 | 0 | 0 | 1 |
| 112 | 1 | 0 | 0 | 0 | 0 | 0 | 0 | 1 |
| 117 | 0 | 0 | 0 | 1 | 0 | 0 | 0 | 1 |
| 125 | 1 | 0 | 0 | 0 | 0 | 0 | 0 | 1 |
| 138 | 1 | 0 | 0 | 0 | 0 | 0 | 0 | 1 |
| 141 | 1 | 0 | 0 | 0 | 0 | 0 | 0 | 1 |
| 164 | 1 | 0 | 0 | 0 | 0 | 0 | 0 | 1 |
| 171 | 1 | 0 | 0 | 0 | 0 | 0 | 0 | 1 |
| 178 | 1 | 0 | 0 | 0 | 0 | 0 | 0 | 1 |
| 180 | 1 | 0 | 0 | 0 | 0 | 0 | 0 | 1 |
| 193 | 1 | 0 | 0 | 0 | 0 | 0 | 0 | 1 |
| 219 | 1 | 0 | 0 | 0 | 0 | 0 | 0 | 1 |
| 220 | 1 | 0 | 0 | 0 | 0 | 0 | 0 | 1 |
| 238 | 0 | 1 | 0 | 0 | 0 | 0 | 0 | 1 |
| 242 | 1 | 0 | 0 | 0 | 0 | 0 | 0 | 1 |
| Closest match: 18 | 1 | 0 | 0 | 0 | 0 | 0 | 0 | 1 |
| Closest match: 270 | 1 | 0 | 0 | 0 | 0 | 0 | 0 | 1 |
| Closest match: 336 | 1 | 0 | 0 | 0 | 0 | 0 | 0 | 1 |
| Closest match: 361 | 0 | 0 | 0 | 1 | 0 | 0 | 0 | 1 |
| Closest match: 4 | 1 | 0 | 0 | 0 | 0 | 0 | 0 | 1 |
| Closest match: 8 | 1 | 0 | 0 | 0 | 0 | 0 | 0 | 1 |
| Closest match: 95 | 0 | 0 | 0 | 0 | 1 | 0 | 0 | 1 |
| Closest match: 18 | 1 | 0 | 0 | 0 | 0 | 0 | 0 | 1 |
| Closest match: 97 | 0 | 1 | 0 | 0 | 0 | 0 | 0 | 1 |
| **All** | **617** | **7** | **22** | **62** | **12** | **6** | **4** | **730** |

Supplementary table 5. Frequency of the determined *ftsI* allele versus capsular type in 730 invasive *H. influenzae* isolates.

| ***ftsI* allele** | **a** | **b** | **c** | **e** | **f** | **Non-capsular** | **Total** |
| --- | --- | --- | --- | --- | --- | --- | --- |
| 10 | 0 | 62 | 0 | 0 | 0 | 72 | 134 |
| 6 | 0 | 0 | 0 | 0 | 101 | 0 | 101 |
| 8 | 0 | 0 | 0 | 0 | 0 | 60 | 60 |
| 27 | 0 | 0 | 1 | 0 | 0 | 59 | 60 |
| 37 | 0 | 0 | 0 | 0 | 0 | 57 | 57 |
| 4 | 0 | 0 | 0 | 0 | 0 | 39 | 39 |
| 1 | 0 | 0 | 0 | 0 | 0 | 25 | 25 |
| 2 | 0 | 2 | 0 | 0 | 0 | 20 | 22 |
| 39 | 0 | 1 | 0 | 0 | 0 | 21 | 22 |
| 43 | 0 | 0 | 0 | 0 | 0 | 21 | 21 |
| 18 | 0 | 0 | 0 | 0 | 0 | 18 | 18 |
| 55 | 0 | 0 | 0 | 15 | 0 | 0 | 15 |
| 34 | 0 | 0 | 0 | 0 | 0 | 13 | 13 |
| 29 | 0 | 0 | 0 | 0 | 0 | 9 | 9 |
| 50 | 0 | 0 | 0 | 0 | 0 | 8 | 8 |
| 63 | 0 | 0 | 0 | 0 | 0 | 8 | 8 |
| 15 | 0 | 0 | 0 | 0 | 0 | 7 | 7 |
| 116 | 0 | 0 | 0 | 0 | 0 | 7 | 7 |
| Closest match: 342 | 0 | 6 | 0 | 0 | 0 | 0 | 6 |
| 12 | 0 | 0 | 0 | 0 | 0 | 5 | 5 |
| 104 | 0 | 0 | 0 | 0 | 0 | 5 | 5 |
| 5 | 0 | 0 | 0 | 0 | 0 | 4 | 4 |
| 23 | 0 | 1 | 0 | 0 | 0 | 3 | 4 |
| 48 | 0 | 0 | 0 | 0 | 0 | 4 | 4 |
| 53 | 0 | 0 | 0 | 0 | 0 | 4 | 4 |
| 97 | 0 | 0 | 0 | 0 | 0 | 4 | 4 |
| 126 | 0 | 0 | 0 | 0 | 0 | 4 | 4 |
| 135 | 0 | 0 | 0 | 0 | 0 | 4 | 4 |
| 46 | 3 | 0 | 0 | 0 | 0 | 0 | 3 |
| 106 | 0 | 0 | 0 | 0 | 0 | 3 | 3 |
| Closest match: 15 | 0 | 0 | 0 | 0 | 0 | 3 | 3 |
| 9 | 0 | 0 | 0 | 0 | 0 | 2 | 2 |
| 20 | 0 | 0 | 0 | 0 | 0 | 2 | 2 |
| 21 | 0 | 0 | 0 | 0 | 0 | 2 | 2 |
| 32 | 0 | 0 | 0 | 0 | 0 | 2 | 2 |
| 70 | 0 | 0 | 0 | 0 | 0 | 2 | 2 |
| 132 | 0 | 0 | 0 | 0 | 0 | 2 | 2 |
| 137 | 0 | 0 | 0 | 0 | 0 | 2 | 2 |
| 156 | 0 | 0 | 0 | 0 | 0 | 2 | 2 |
| 214 | 0 | 0 | 0 | 0 | 0 | 2 | 2 |
| 13 | 0 | 0 | 0 | 0 | 0 | 1 | 1 |
| 17 | 0 | 0 | 0 | 0 | 0 | 1 | 1 |
| 31 | 0 | 0 | 0 | 0 | 0 | 1 | 1 |
| 33 | 0 | 0 | 0 | 0 | 0 | 1 | 1 |
| 35 | 0 | 0 | 0 | 0 | 0 | 1 | 1 |
| 38 | 0 | 0 | 0 | 0 | 0 | 1 | 1 |
| 52 | 0 | 0 | 0 | 0 | 0 | 1 | 1 |
| 56 | 0 | 0 | 0 | 0 | 0 | 1 | 1 |
| 75 | 0 | 0 | 0 | 0 | 0 | 1 | 1 |
| 111 | 0 | 0 | 0 | 0 | 0 | 1 | 1 |
| 112 | 0 | 0 | 0 | 0 | 0 | 1 | 1 |
| 117 | 0 | 0 | 0 | 0 | 0 | 1 | 1 |
| 125 | 0 | 0 | 0 | 0 | 0 | 1 | 1 |
| 138 | 0 | 0 | 0 | 0 | 0 | 1 | 1 |
| 141 | 0 | 0 | 0 | 0 | 0 | 1 | 1 |
| 164 | 0 | 0 | 0 | 0 | 0 | 1 | 1 |
| 171 | 0 | 0 | 0 | 0 | 0 | 1 | 1 |
| 178 | 0 | 0 | 0 | 0 | 0 | 1 | 1 |
| 180 | 0 | 0 | 0 | 0 | 0 | 1 | 1 |
| 193 | 0 | 0 | 0 | 0 | 0 | 1 | 1 |
| 219 | 0 | 0 | 0 | 0 | 0 | 1 | 1 |
| 220 | 0 | 0 | 0 | 0 | 0 | 1 | 1 |
| 238 | 0 | 0 | 0 | 0 | 0 | 1 | 1 |
| 242 | 0 | 0 | 0 | 0 | 0 | 1 | 1 |
| Closest match: 18 | 0 | 0 | 0 | 0 | 0 | 1 | 1 |
| Closest match: 270 | 0 | 0 | 0 | 0 | 1 | 0 | 1 |
| Closest match: 336 | 0 | 0 | 0 | 0 | 0 | 1 | 1 |
| Closest match: 361 | 0 | 0 | 0 | 0 | 0 | 1 | 1 |
| Closest match: 4 | 0 | 0 | 0 | 0 | 0 | 1 | 1 |
| Closest match: 8 | 0 | 0 | 0 | 0 | 0 | 1 | 1 |
| Closest match: 95 | 0 | 0 | 0 | 0 | 0 | 1 | 1 |
| Closest match: 18 | 0 | 0 | 0 | 0 | 0 | 1 | 1 |
| Closest match: 97 | 0 | 0 | 0 | 0 | 0 | 1 | 1 |
| **All** | **3** | **72** | **1** | **15** | **102** | **537** | **730** |

Supplementary table 6: MLST Cloncal complex versus PBP3 group

|  |  | **PBP3 group** | | | | | |  |
| --- | --- | --- | --- | --- | --- | --- | --- | --- |
| **MLST Cloncal complex** | **No mutation** | **I** | **IIa** | **IIb** | **IIc** | **IId** | **III like** | **Total** |
| ST-124 complex | 102 | 0 | 0 | 0 | 0 | 0 | 0 | 102 |
| ST-3 complex | 41 | 3 | 0 | 35 | 0 | 0 | 2 | 81 |
| Not applicable | 73 | 0 | 0 | 4 | 1 | 0 | 0 | 78 |
| ST-6 complex | 62 | 0 | 0 | 3 | 6 | 0 | 0 | 71 |
| ST-11 complex | 61 | 0 | 0 | 0 | 0 | 2 | 1 | 64 |
| ST-107 complex | 14 | 4 | 19 | 1 | 1 | 0 | 0 | 39 |
| ST-836 complex | 25 | 0 | 0 | 0 | 1 | 0 | 1 | 27 |
| ST-12 complex | 18 | 0 | 0 | 3 | 0 | 0 | 0 | 21 |
| ST-18 complex | 14 | 0 | 0 | 1 | 0 | 0 | 0 | 15 |
| ST-165 complex | 9 | 0 | 0 | 5 | 0 | 0 | 0 | 14 |
| ST-425 complex | 14 | 0 | 0 | 0 | 0 | 0 | 0 | 14 |
| ST-105 complex | 11 | 0 | 0 | 0 | 0 | 0 | 0 | 11 |
| ST-210 complex | 11 | 0 | 0 | 0 | 0 | 0 | 0 | 11 |
| ST-57 complex | 9 | 0 | 0 | 2 | 0 | 0 | 0 | 11 |
| ST-584 complex | 11 | 0 | 0 | 0 | 0 | 0 | 0 | 11 |
| ST-266 complex | 8 | 0 | 0 | 0 | 2 | 0 | 0 | 10 |
| ST-472 complex | 10 | 0 | 0 | 0 | 0 | 0 | 0 | 10 |
| ST-139 complex | 8 | 0 | 0 | 1 | 0 | 0 | 0 | 9 |
| ST-422 complex | 5 | 0 | 1 | 3 | 0 | 0 | 0 | 9 |
| ST-199 complex | 8 | 0 | 0 | 0 | 0 | 0 | 0 | 8 |
| ST-155 complex | 7 | 0 | 0 | 0 | 0 | 0 | 0 | 7 |
| ST-183 complex | 6 | 0 | 0 | 1 | 0 | 0 | 0 | 7 |
| ST-264 complex | 6 | 0 | 0 | 1 | 0 | 0 | 0 | 7 |
| ST-395 complex | 5 | 0 | 0 | 2 | 0 | 0 | 0 | 7 |
| ST-84 complex | 7 | 0 | 0 | 0 | 0 | 0 | 0 | 7 |
| ST-1426 complex | 6 | 0 | 0 | 0 | 0 | 0 | 0 | 6 |
| ST-393 complex | 6 | 0 | 0 | 0 | 0 | 0 | 0 | 6 |
| ST-652 complex | 6 | 0 | 0 | 0 | 0 | 0 | 0 | 6 |
| ST-1836 complex | 5 | 0 | 0 | 0 | 0 | 0 | 0 | 5 |
| ST-34 complex | 5 | 0 | 0 | 0 | 0 | 0 | 0 | 5 |
| ST-41 complex | 5 | 0 | 0 | 0 | 0 | 0 | 0 | 5 |
| ST-746 complex | 5 | 0 | 0 | 0 | 0 | 0 | 0 | 5 |
| ST-142 complex | 2 | 0 | 2 | 0 | 0 | 0 | 0 | 4 |
| ST-321 complex | 4 | 0 | 0 | 0 | 0 | 0 | 0 | 4 |
| ST-389 complex | 4 | 0 | 0 | 0 | 0 | 0 | 0 | 4 |
| ST-396 complex | 0 | 0 | 0 | 0 | 0 | 4 | 0 | 4 |
| ST-992 complex | 4 | 0 | 0 | 0 | 0 | 0 | 0 | 4 |
| ST-163 complex | 3 | 0 | 0 | 0 | 0 | 0 | 0 | 3 |
| ST-23 complex | 3 | 0 | 0 | 0 | 0 | 0 | 0 | 3 |
| ST-519 complex | 3 | 0 | 0 | 0 | 0 | 0 | 0 | 3 |
| ST-474 complex | 2 | 0 | 0 | 0 | 0 | 0 | 0 | 2 |
| ST-487 complex | 2 | 0 | 0 | 0 | 0 | 0 | 0 | 2 |
| ST-931 complex | 2 | 0 | 0 | 0 | 0 | 0 | 0 | 2 |
| ST-1025 complex | 0 | 0 | 0 | 0 | 1 | 0 | 0 | 1 |
| ST-1477 complex | 1 | 0 | 0 | 0 | 0 | 0 | 0 | 1 |
| ST-1529 complex | 1 | 0 | 0 | 0 | 0 | 0 | 0 | 1 |
| ST-249 complex | 1 | 0 | 0 | 0 | 0 | 0 | 0 | 1 |
| ST-513 complex | 1 | 0 | 0 | 0 | 0 | 0 | 0 | 1 |
| ST-589 complex | 1 | 0 | 0 | 0 | 0 | 0 | 0 | 1 |
| **All** | **617** | **7** | **22** | **62** | **12** | **6** | **4** | **730** |

Supplementary table 7. Frequency of the determined *ftsI* allele versus genetic beta-lactam resistance mechanism in 730 invasive *H. influenzae* isolates.

|  | **Genetic beta-lactam resistance mechanism** | | | |  |
| --- | --- | --- | --- | --- | --- |
| ***ftsI* allele** | **gBLNAS** | **gBLPAR** | **gBLNAR** | **gBLPACR** | **Total** |
| 10 | 122 | 12 | 0 | 0 | 134 |
| 6 | 100 | 1 | 0 | 0 | 101 |
| 8 | 53 | 6 | 1 | 0 | 60 |
| 27 | 30 | 29 | 1 | 0 | 60 |
| 37 | 54 | 3 | 0 | 0 | 57 |
| 4 | 32 | 7 | 0 | 0 | 39 |
| 1 | 1 | 0 | 19 | 5 | 25 |
| 2 | 0 | 0 | 21 | 1 | 22 |
| 39 | 22 | 0 | 0 | 0 | 22 |
| 43 | 1 | 0 | 19 | 1 | 21 |
| 18 | 18 | 0 | 0 | 0 | 18 |
| 55 | 14 | 0 | 1 | 0 | 15 |
| 34 | 13 | 0 | 0 | 0 | 13 |
| 29 | 0 | 9 | 0 | 0 | 9 |
| 50 | 8 | 0 | 0 | 0 | 8 |
| 63 | 8 | 0 | 0 | 0 | 8 |
| 15 | 4 | 3 | 0 | 0 | 7 |
| 116 | 7 | 0 | 0 | 0 | 7 |
| Closest match: 342 | 0 | 0 | 0 | 6 | 6 |
| 12 | 1 | 4 | 0 | 0 | 5 |
| 104 | 4 | 1 | 0 | 0 | 5 |
| 5 | 0 | 0 | 4 | 0 | 4 |
| 23 | 0 | 0 | 4 | 0 | 4 |
| 48 | 0 | 0 | 4 | 0 | 4 |
| 53 | 4 | 0 | 0 | 0 | 4 |
| 97 | 0 | 0 | 4 | 0 | 4 |
| 126 | 2 | 2 | 0 | 0 | 4 |
| 135 | 4 | 0 | 0 | 0 | 4 |
| 46 | 3 | 0 | 0 | 0 | 3 |
| 106 | 3 | 0 | 0 | 0 | 3 |
| Closest match: 15 | 3 | 0 | 0 | 0 | 3 |
| 9 | 0 | 0 | 2 | 0 | 2 |
| 20 | 0 | 0 | 2 | 0 | 2 |
| 21 | 0 | 0 | 1 | 1 | 2 |
| 32 | 0 | 0 | 2 | 0 | 2 |
| 70 | 2 | 0 | 0 | 0 | 2 |
| 132 | 2 | 0 | 0 | 0 | 2 |
| 137 | 2 | 0 | 0 | 0 | 2 |
| 156 | 2 | 0 | 0 | 0 | 2 |
| 214 | 0 | 0 | 2 | 0 | 2 |
| 13 | 0 | 0 | 1 | 0 | 1 |
| 17 | 0 | 0 | 1 | 0 | 1 |
| 31 | 1 | 0 | 0 | 0 | 1 |
| 33 | 0 | 0 | 0 | 1 | 1 |
| 35 | 0 | 0 | 1 | 0 | 1 |
| 38 | 0 | 0 | 1 | 0 | 1 |
| 52 | 0 | 0 | 1 | 0 | 1 |
| 56 | 1 | 0 | 0 | 0 | 1 |
| 75 | 0 | 0 | 1 | 0 | 1 |
| 111 | 1 | 0 | 0 | 0 | 1 |
| 112 | 1 | 0 | 0 | 0 | 1 |
| 117 | 0 | 0 | 1 | 0 | 1 |
| 125 | 1 | 0 | 0 | 0 | 1 |
| 138 | 1 | 0 | 0 | 0 | 1 |
| 141 | 1 | 0 | 0 | 0 | 1 |
| 164 | 1 | 0 | 0 | 0 | 1 |
| 171 | 1 | 0 | 0 | 0 | 1 |
| 178 | 1 | 0 | 0 | 0 | 1 |
| 180 | 1 | 0 | 0 | 0 | 1 |
| 193 | 1 | 0 | 0 | 0 | 1 |
| 219 | 1 | 0 | 0 | 0 | 1 |
| 220 | 1 | 0 | 0 | 0 | 1 |
| 238 | 0 | 0 | 1 | 0 | 1 |
| 242 | 1 | 0 | 0 | 0 | 1 |
| Closest match: 18 | 1 | 0 | 0 | 0 | 1 |
| Closest match: 270 | 1 | 0 | 0 | 0 | 1 |
| Closest match: 336 | 1 | 0 | 0 | 0 | 1 |
| Closest match: 361 | 0 | 0 | 1 | 0 | 1 |
| Closest match: 4 | 1 | 0 | 0 | 0 | 1 |
| Closest match: 8 | 1 | 0 | 0 | 0 | 1 |
| Closest match: 95 | 0 | 0 | 1 | 0 | 1 |
| Closest match: 18 | 1 | 0 | 0 | 0 | 1 |
| Closest match: 97 | 0 | 0 | 1 | 0 | 1 |
| **All** | **540** | **77** | **98** | **15** | **730** |

Supplementary table 8. Amino acid substitutions in different PBP3 subtypes for BLNAR and BLPACR strains compared with Rd control.

| Amino acid substitution | 311 | 337 | 350 | 352 | 357 | 368 | 377 | 385 | 389 | 437 | 443 | 449 | 490 | 501 | 502 | 511 | 517 | 526 | 528 | 530 | 532 | 547 | 562 | 569 | 586 |
| --- | --- | --- | --- | --- | --- | --- | --- | --- | --- | --- | --- | --- | --- | --- | --- | --- | --- | --- | --- | --- | --- | --- | --- | --- | --- |
| Rd control | S | A | D | S | S | A | M | S | L | A | T | I | G | R | A | V | R | N | Y | A | T | V | V | N | A |
| I |  |  |  |  |  |  |  |  |  |  |  |  |  |  |  |  | H |  |  |  |  |  |  |  |  |
| IIa |  |  |  |  |  |  |  |  |  |  |  |  |  |  |  |  |  | K |  |  |  |  |  |  |  |
| IIb |  |  |  |  |  |  |  |  |  |  |  |  |  |  | V |  |  | K |  |  |  |  |  |  |  |
| IIc |  |  |  |  |  |  |  |  |  |  |  |  |  |  | T |  |  | K |  |  |  |  |  |  |  |
| IId |  |  |  |  |  |  |  |  |  |  |  | V |  |  |  |  |  | K |  |  |  |  |  |  |  |
| III |  |  |  |  |  |  |  | T |  |  |  |  |  |  |  |  |  | K |  |  |  |  |  |  |  |
| III+ |  |  |  |  |  |  |  | T | F |  |  |  |  |  |  |  |  | K |  |  |  |  |  |  |  |
| III like |  |  |  |  |  |  |  | T |  |  |  |  |  |  |  |  | H |  |  |  |  |  |  |  |  |
| III like + |  |  |  |  |  |  |  | T | F |  |  |  |  |  |  |  | H |  |  |  |  |  |  |  |  |
| Polymorphic | P | V/N | N | T/N/F | N | T | I | T | F | S | A | V | E | E/L/H | T/V | A | H | K | H | S | S | I | L | S | S |

Supplementary table 9. Other resistance genes detected in 730 invasive *H. influenzae* isolates.

| **Resistance gene** | **Number of isolates** | **Drug target** |
| --- | --- | --- |
| *aph(3'')-Ib* | 14 | Aminoglycoside |
| *aph(3')-Ia* | 13 | Aminoglycoside |
| *aph(6)-Id* | 12 | Aminoglycoside |
| *tet(B)* | 8 | Tetracycline |
| *sul2* | 6 | Sulfonamide |
| *mef(A)* | 1 | Macrolide |
| *msr(D)* | 1 | Macrolide |
